# Between-area communication through the lens of within-area neuronal dynamics

**DOI:** 10.1126/sciadv.adl6120

**Published:** 2024-10-16

**Authors:** Olivia Gozel, Brent Doiron

**Affiliations:** ^1^ Departments of Neurobiology and Statistics, University of Chicago, Chicago, IL 60637, USA.; ^2^ Grossman Center for Quantitative Biology and Human Behavior, University of Chicago, Chicago, IL 60637, USA.

## Abstract

A core problem in systems and circuits neuroscience is deciphering the origin of shared dynamics in neuronal activity: Do they emerge through local network interactions, or are they inherited from external sources? We explore this question with large-scale networks of spatially ordered spiking neuron models where a downstream network receives input from an upstream sender network. We show that linear measures of the communication between the sender and receiver networks can discriminate between emergent or inherited population dynamics. A match in the dimensionality of the sender and receiver population activities promotes faithful communication. In contrast, a nonlinear mapping between the sender to receiver activity, for example, through downstream emergent population-wide fluctuations, can impair linear communication. Our work exposes the benefits and limitations of linear measures when analyzing between-area communication in circuits with rich population-wide neuronal dynamics.

## Teaser

Linear communication measures can discriminate between emergent and inherited population dynamics.

## INTRODUCTION

The brain is composed of a multitude of distributed areas that interact to support the complex computations needed for perception and cognition. While past experimental investigations were typically limited to single-neuron recordings, recent technological advances allow for sampling from large populations of neurons simultaneously (*1*). This newfound ability was initially used to characterize the local dynamics of a population of neurons from the same brain area (*2*–*6*). Presently, many studies measure population activity distributed over several brain regions, giving a more holistic, brain-wide view of neuronal processing (*7*–*9*). However, despite these richer datasets, the science of the mechanics by which different brain areas communicate with one another is still in its infancy.

An often used measure of neuron-to-neuron interaction is the joint trial-to-trial covariability, or noise correlation, of their spike train responses (*10*, *11*). The idea is that neuron pairs that have high noise correlation are likely members of the same putative neuronal circuit (*12*–*14*). While pairwise correlations can be informative (*11*), the large-scale nature of population recordings presents a challenge when attempting to expose the salient aspects of population-wide interactions simply from an analysis of neuron pairs (*15*). Dimensionality reduction techniques have been developed to frame population activity within a space of the appropriate size: Large enough to capture the core shared variability across a population, yet small enough to be tractable (*4*, *16*). These analysis techniques identify low-dimensional structure in the population-wide activity, and recent work has used them to measure how connected brain areas interact with one another (*17*, *18*). That said, these techniques do not, on their own, provide insight into the circuit mechanisms that support or impede brain area–to–brain area communication.

The propagation of brain activity has been the focus of extensive circuit modeling attempts. Feedforward networks are the base structure of many contemporary models of object classification and have been used with great success to model the performance of visual system hierarchy (*19*). However, networks of spiking neuron models with random, sparse feedforward connectivity produce propagation that leads to excessive, often rhythmic, synchronization (*12*, *20*–*23*). By contrast, a single population of spiking neuron models with sparse, yet strong, excitatory and inhibitory recurrent connections can show temporally irregular, roughly asynchronous spiking dynamics (*24*–*27*), mimicking what is often considered the default state of cortical networks (*13*, *26*). Neurophysiological recordings over a range of sensory and cognitive states show a wide distribution of spike count correlations whose average is low but positive and notably different from zero (*10*, *11*), in disagreement with the asynchronous activity of classical models. Recent modeling work shows how population dynamics with stable firing rates yet moderate population-wide noise correlations can be produced when structured synaptic wiring is considered, such as discrete block structure (*28*), low-rank recurrent components (*29*, *30*), or distance-dependent connection probability (*27*, *31*, *32*). While these results provide insights into how circuit structure determines shared variability, they have been restricted to within-population dynamics. On the other hand, recent modeling efforts have shed some light on the interaction between brain areas (*33*–*35*), yet often without a consideration of response fluctuations. Thus, there remains a gap in understanding how the circuit-based theories of shared variability within a population extend to the distribution (or propagation) of variability between populations.

In this work, we investigate how complex within brain-area neuronal dynamics affect interactions between distinct brain areas using a network of model spiking neurons with biologically plausible synaptic connectivity and dynamics. We determine conditions when communication is disrupted between an upstream sender network and a downstream receiver network, as assessed by linear measures. We show that the emergence of complex spatiotemporal dynamics within the sender network leads to faithful sender-receiver communication, while if the receiver generates complex dynamics, then communication is disrupted. We understand this dichotomy through how shared fluctuations in the receiver align or misalign with respect to the fluctuations in the sender. Last, when sender-receiver linear communication is disrupted, it occurs in one of two ways: by inducing a nonlinear mapping of the sender-receiver activity or by yielding chaotic spiking dynamics at the macroscopic scale in the receiver. These results expose the limitations of linear measures when deciphering brain-area communication in the presence of complex spatiotemporal neuronal dynamics.

## RESULTS

### Destabilization of E/I balance yields rich within-area population-wide dynamics

**Fig. 1.**
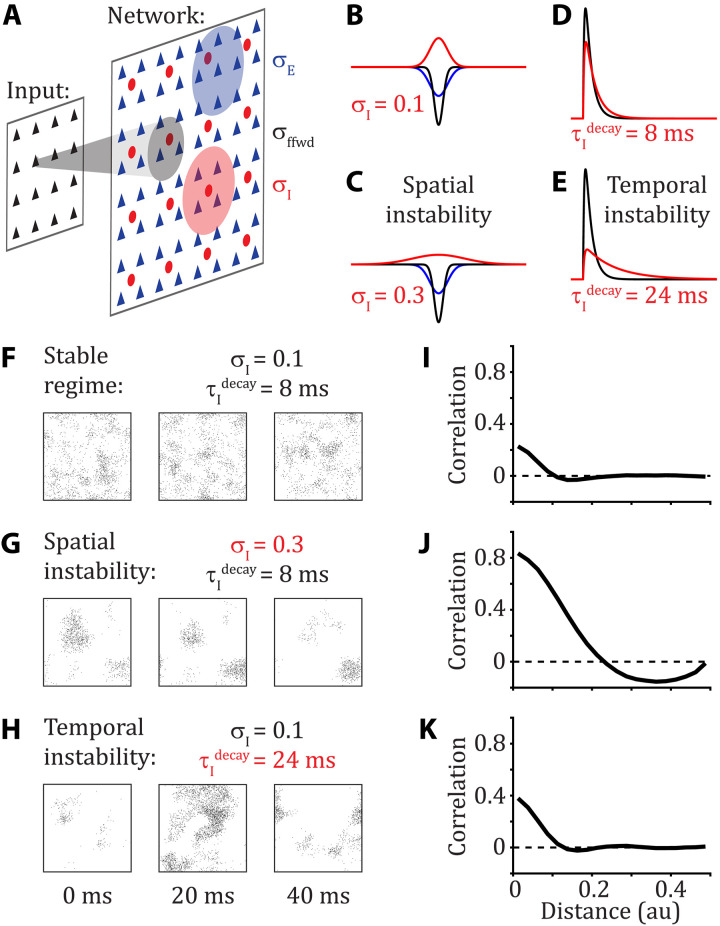
Destabilization of E/I balance yields rich within-area population-wide dynamics. (**A**) The input layer (black triangles) produces temporally and spatially homogeneous, independent Poisson spike trains. The input layer connects to a network with excitatory (E; blue triangles) and inhibitory (I; red discs) spiking neuron models that are recurrently connected to one another. The neurons are arranged on a two-dimensional [0,1] × [0,1] grid. Connections are spatially organized according to a wrapped Gaussian (periodic boundary conditions) with widths σ_ffwd_, σ_E_, and σ_I_ for the feedforward and recurrent E and I connections, respectively (standard parameters: σ_ffwd_ = 0.05 and σ_E_ = 0.1). (**B** and **C**) Connection probability as a function of distance from feedforward input (black), recurrent excitation (blue), and recurrent inhibition (red). In the standard network, recurrent inhibitory connections balance excitatory connections to yield a stable regime (B) (σ_I_ = 0.1). When recurrent inhibitory connections are broadened (C) (σ_I_ = 0.3), E/I balance is spatially destabilized. (**D** and **E**) Excitatory postsynaptic potential (black) and inhibitory postsynaptic potential (red). In the standard network, the time courses of excitatory and inhibitory postsynaptic potentials balance each other to yield a stable regime (D) ($\tau_{I}^{\text{decay}}=8$ ms). When the time constant of inhibitory neurons is increased (E) ($\tau_{I}^{\text{decay}}=24$ ms), E/I balance is temporally destabilized. (**F** to **H**) Raster plot snapshots of the spiking activity in the recurrent network over 2-ms time windows separated by 20 ms: (F) standard network in the stable regime, (G) spatial instability induced by lateral inhibition, and (H) temporal instability induced by slow inhibition. (**I** to **K**) Corresponding pairwise correlation as a function of pairwise distance. au, arbitrary units.

Before we explore communication between brain areas, we first discuss how within brain-area neuronal dynamics depend on the temporal and structural makeup of the recurrent synaptic interactions between neurons. We use a layered network of spiking neuron models that are spatially organized on a square grid (Fig. 1A and see the “Network structure” section). Neurons in the input layer are modeled as independent homogeneous Poisson processes with a uniform rate. They project their activity to a recurrently coupled network of excitatory (E) and inhibitory (I) spiking neuron models (exponential integrate and fire; see the “Neuronal dynamics” section). For all analyses, instantaneous firing rates were computed from the spike counts in nonoverlapping 50-ms bins.

Throughout our study, the spatial and temporal scales of synaptic interactions are key parameters. Synaptic currents are modeled as a difference of exponentials with rise and decay timescales, τ^rise^ and τ^decay^, respectively. It is well known that synaptic connectivity is spatially structured with connection probabilities falling off with the distance between pre- and postsynaptic neurons (*36*–*38*). Accordingly, following past works (*27*, *32*, *39*), we model within- and between-layer connectivity as obeying a two-dimensional Gaussian whose spatial widths are denoted by σ (see the “Network structure” section) and assume periodic boundary conditions on our domain. We set a larger width for the recurrent than the feedforward connections (σ_rec_ > σ_ffwd_) because these networks exhibit correlated neuronal spiking dynamics (*27*).

Unless otherwise specified, our network is set with the parameters reported in Table 1. We call these the standard parameters (Fig. 1, B and D) because they yield stable spiking dynamics, as reflected by temporally irregular spiking activity (Fig. 1F) and an average pairwise spike-count correlation over all spatial scales close to zero (Fig. 1I) (*27*). It is known from previous work that when the E/I network parameters lead to destabilization of firing rate dynamics, spatiotemporal patterns of spiking activity intrinsically emerge within the network (*31*, *32*, *34*). E/I balance can either be destabilized in space by increasing the width of recurrent inhibition σ_I_ (Fig. 1C), or in time by increasing the inhibitory synaptic time constant $\tau_{I}^{\text{decay}}$ (Fig. 1E). The spatiotemporal characteristics of the emerging patterns of activity within the recurrent network depend on the route (spatial or temporal) to E/I destabilization. If E/I balance is spatially destabilized, then it yields spatially organized patterns of activity whose spatial scale depends on the width of recurrent inhibition (Fig. 1G). It has the effect to increase the pairwise spike-count correlations for short pairwise distances, while yielding negative correlations at broader distances (Fig. 1J). Alternatively, if E/I balance is temporally destabilized, then it yields temporally organized patterns of activity that propagate across the entire network (Fig. 1H). Contrary to a spatial destabilization, the pairwise spike-count correlations are only slightly increased at short pairwise distances (Fig. 1K). In sum, this modeling framework gives us control of the emergence and structure of complex, population-wide spiking dynamics in the recurrent network without the need for a structured external source of noise. We next investigate whether and how complex within-area neuronal dynamics affect the communication between brain areas.

**Table 1.** Standard parameters for the simulations. The synaptic strength parameters (*j*) are scaled by 1/√*N* in the voltage dynamics (Eqs. 3 and 4), so that PSPs are in reasonable experimental ranges (i.e < 1 mV).

Network connectivity	σ_ffwd_ = 0.05	σ_rec_ = 0.1	σ_E_ = 0.1	σ_I_ = 0.1
	p¯E ffwd=0.1	${\bar{p}}_{I}^{\text{ffwd}}=0.05$	$j_{E}^{\text{ffwd}}=240$	$j_{I}^{\text{ffwd}}=400$
	${\bar{p}}_{\text{EE}}^{\text{rec}}=0.01$	${\bar{p}}_{\text{EI}}^{\text{rec}}=0.04$	${\bar{p}}_{\text{IE}}^{\text{rec}}=0.03$	${\bar{p}}_{\text{II}}^{\text{rec}}=0.04$
	$j_{\text{EE}}^{\text{rec}}=80\;\mathrm{mV}$	$j_{\text{EI}}^{\text{rec}}=-240\;\mathrm{mV}$	$j_{\text{IE}}^{\text{rec}}=40\;\mathrm{mV}$	$j_{\text{II}}^{\text{rec}}=-300\;\mathrm{mV}$
Neuronal dynamics	*C*_m_ = 1 ms	$g_{L}^{E}=1/15$	$g_{L}^{I}=1/10$	*V*_L_ =−60 mV
	*V*_th_ =−10 mV	*V*_reset_ =−65 mV	$t_{\text{ref}}^{E}=1.5$ ms	$t_{\text{ref}}^{I}=0.5$ ms
	*V*_T_ =−50 mV	$\Delta_{T}^{E}=2$ mV	$\Delta_{T}^{I}=0.5$ mV	
	$\tau_{E}^{\text{rise}}=1$ ms	$\tau_{E}^{\text{decay}}=5$ ms	$\tau_{I}^{\text{rise}}=1$ ms	$\tau_{I}^{\text{decay}}=8$ ms
Average firing rate	Standard network		${\bar{\nu }}^{\left(S\right)}\approx 20$ Hz	${\bar{\nu }}^{\left(R\right)}\approx 35$ Hz
	Spatially destabilized network ($\sigma_{I}^{\left(S\right)}=0.3$)		${\bar{\nu }}^{\left(S\right)}\approx 12$ Hz	${\bar{\nu }}^{\left(R\right)}\approx 17$ Hz
	Spatially destabilized network ($\sigma_{I}^{\left(R\right)}=0.3$)		${\bar{\nu }}^{\left(S\right)}\approx 20$ Hz	${\bar{\nu }}^{\left(R\right)}\approx 22$ Hz
	Temporally destabilized network ($\tau_{I}^{\text{decay}\left(S\right)}=24$ ms)		${\bar{\nu }}^{\left(S\right)}\approx 16$ Hz	${\bar{\nu }}^{\left(R\right)}\approx 24$ Hz
	Temporally destabilized network ($\tau_{I}^{\text{decay}\left(R\right)}=24$ ms)		${\bar{\nu }}^{\left(S\right)}\approx 20$ Hz	${\bar{\nu }}^{\left(R\right)}\approx 28$ Hz

### Between-area spike count correlations are differentially affected by the location and type of destabilization of the E/I balance

**Fig. 2.**
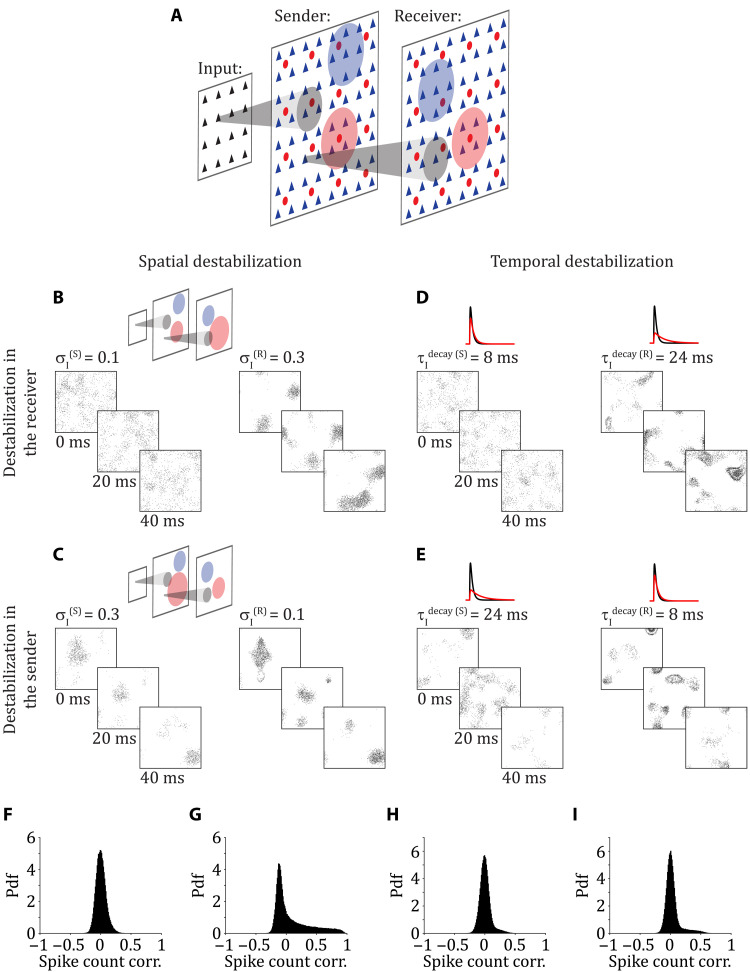
Between-area spike count correlations are differentially affected by the location and type of destabilization of the E/I balance. (**A**) The input layer produces homogeneous Poisson spike trains and connects to the sender layer (S), which itself connects to the receiver layer (R). (**B** and **C**) Simultaneous rasters of activity in sender (left) and receiver (right) layers when E/I balance is spatially destabilized by increasing the width of recurrent inhibitory connections, σ_I_, in the receiver (B) or in the sender (C). (**D** and **E**) Simultaneous rasters of activity in the sender (left) and the receiver (right) layers when E/I balance is temporally destabilized by increasing the time constant of inhibitory neurons, $\tau_{I}^{\text{decay}}$, in the receiver (D) or in the sender (E). (**F** and **G**) Probability density function (Pdf) of the pairwise spike count correlations between a neuron in the sender and a neuron in the receiver during spatial destabilization in the receiver (F) (mean of the distribution, μ = 0.0061) or in the sender (G) (μ = 0.0846). (**H** and **I**) Probability density function of the pairwise spike count correlations between a neuron in the sender and a neuron in the receiver during temporal destabilization in the receiver (H) (μ = 0.0052) or in the sender (I) (μ = 0.0304).

We extend our framework to next explore the responses of a three-layer network of spiking neuron models (Fig. 2A). As before, neurons in the input layer are modeled as independent homogeneous Poisson processes with a uniform rate. The sender (second layer) and receiver (third layer) populations each consist of excitatory (E) and inhibitory (I) spiking neuron models that are spatially organized on a square grid (see the “Network structure” section). The sender layer projects excitatory connections to the receiver layer, while projections from the receiver to the sender are omitted.

The sender network with standard parameters produces spiking activity that is temporally irregular (Fig. 2, B and D, left), with a near symmetric distribution of pairwise correlations having a mean close to zero (fig. S1, A and C, left). Emergence of spatiotemporal patterns in the receiving network through a spatial destabilization of E/I balance (Fig. 2B, right) induces a broader distribution of spike count correlations with a heavy positive tail (fig. S1A, right). However, the distribution of pairwise correlations between neurons from the sending and receiving networks remains relatively narrow with a mean close to zero (Fig. 2F). By contrast, when E/I balance is instead spatially destabilized in the sender network, it yields the emergence of spatiotemporal patterns that are propagated to the receiving network (Fig. 2C). Consequently, the distribution of spike count correlations is heavily tailed in both the sender and receiver networks (fig. S1B). Furthermore, we observe a heavy tail in the distribution of between-area spike count correlations (Fig. 2G).

When E/I balance is destabilized temporally by increasing the time constant of inhibitory synaptic currents, it yields temporally organized patterns of activity. When these patterns originate in the sender network, they then propagate downstream to the receiver network (Fig. 2E). Alternatively, when the receiver network is temporally destabilized, then these patterns occur only in the receiver (Fig. 2D). Despite this patterning, the within-area pairwise spike count correlations increase only slightly (fig. S1, C and D). Further, the between-area spike count correlations also stay low no matter the location (sender or receiver) of the emergence of spatiotemporal patterns (Fig. 2, H and I). These observations emphasize the diverse characteristics of spatiotemporal population spiking dynamics that depend on the location of their emergence (sender versus receiver) and the mechanisms through which they are induced (spatial versus temporal). However, in all networks, the correlation statistics are close to the experimental literature (*10*, *18*). Distributions of pairwise correlations are broad with a mean close to zero but positive, both within-area (fig. S1) and between-area (Fig. 2, F to I). We report the average firing rates of sender and receiver networks for all scenarios in Table 1.

### Location and characteristics of spatiotemporal dynamics determine within-area dimensionality

While the average pairwise spike count correlation can be a signature of the dynamical regime of a network (*40*), we take advantage of our access to large amounts of synthetic data to move beyond statistics on only bulk pairwise neuronal activity. In this section, we investigate the structure of population-wide shared neuronal variability and how it depends on E/I destabilization.

**Fig. 3.**
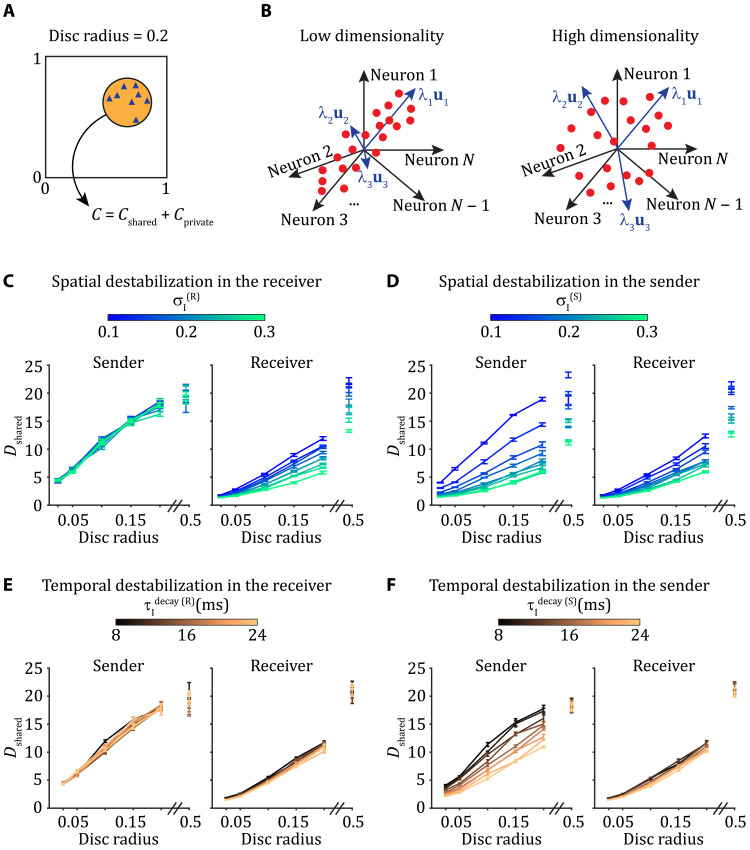
Location and characteristics of spatiotemporal dynamics determine within-area dimensionality. (**A**) Fifty neurons are selected from the neuronal grid by random sampling from discs with different radii. We compute the full covariance matrix of all pairs of selected neurons over time for each individual simulation, *C*, and then obtain the shared covariance matrix through FA. (**B**) Within-area shared dimensionality, *D*_shared_, is measured from the eigenspectrum of *C*_shared_. When the eigenvalues are heterogeneous in magnitude dimensionality is low, whereas when eigenvalues are more uniform in magnitude dimensionality is high. (**C** and **D**) Shared dimensionality as a function of the radius of the disc from which neurons are sampled, either from the sender (left) or from the receiver (right), when we destabilize network activity spatially by modifying σ_I_ in the receiver (C) or in the sender (D). We note that a disc radius of 0.5 indicates a spatial domain that encompasses the full unit square. (**E** and **F**) Same as (C) and (D), when we destabilize the network activity temporally by increasing $\tau_{I}^{\text{decay}}$ in the receiver (E) or in the sender (F).

We randomly select 50 neurons from a local portion of the grid delineated by a disc whose center is also randomly picked (Fig. 3A). By changing the radius of the disc, we explore how within-area shared variability depends on the spatial scale of the sampled population. We only select neurons whose average firing rate is sufficiently responsive (above 2 Hz) and compute their full spike count covariance matrix *C*. Through factor analysis (FA) (*41*, *42*), we separate *C* into a shared component, *C*_shared_, and a private component, *C*_private_ (Fig. 3A and see the “Factor analysis” section). FA, in contrast to probabilistic principal component analysis, does not assume isotropic noise. Hence, the elements of the diagonal matrix *C*_private_ are not constrained to be identical. FA thus determines the directions of the highest covariances and not the largest individual variances as in probabilistic principal component analysis. Singular value decomposition is then applied to *C*_shared_ to obtain the shared eigenvectors and associated eigenvalues that characterize the structure of the shared fluctuations.

While the full distribution of the eigenvalues {λ*_i_*} of the shared covariance matrix *C*_shared_ is informative (figs. S2 and S3), we want to describe this distribution with a single scalar measure. To this end, we compute the effective dimension of within-area shared variability from {λ*_i_*} via $D_{\text{shared}}={\left(\sum \lambda_{i}\right)}^{2}/\sum \lambda_{i}^{2}$, which is sometimes termed the participation ratio (*43*, *44*). Contrary to other measures of dimensionality (*17*, *45*–*47*), *D*_shared_ does not require an arbitrary threshold to give an integer value of dimension. Rather, *D*_shared_ is the squared first moment of the eigenspectrum normalized by the second moment. If the shared fluctuations preferentially take place over a few dimensions, as reflected by a few eigenvalues, λ*_i_*, which are much larger than the others, then it yields low dimensionality *D*_shared_ (Fig. 3B, left). On the other hand, if the shared fluctuations are broadly distributed over the whole eigenspace, as reflected by a uniform distribution of the eigenvalues, then the resulting dimensionality *D*_shared_ is high (Fig. 3B, right). Using within-area shared dimensionality *D*_shared_, we investigate how the emergence of spatiotemporal patterns affects the structure of shared variability, both within-area and in the interaction between connected areas.

In the network with standard parameters, as the disc radius over which neurons are sampled increases, the estimated within-area shared dimensionality *D*_shared_ increases (Fig. 3, C and E, left). This is expected given how the dominant eigenmode changes with disc radius (fig. S2, A and C). However, when sampling the 50 neurons from the whole grid (disc radius = 0.5), the within-area shared dimensionality stays much lower than the theoretical upper bound of 50 that would correspond to a scenario with fully independent and statistically identical neurons (Fig. 3, C and E, left). Because dimensionality depends strongly on the spatial arrangement of the sampled neurons, we cannot simply associate a unique dimensionality value to the network dynamics as a whole. Instead, we focus on how changes in network dynamics owing to E/I destabilization are reflected in changes in dimensionality.

In the network with standard parameters yielding stable activity in both the sender and the receiver networks, the dimensionality decreases (at fixed disc radius) as activity propagates from the sender to the receiver [$\sigma_{I}^{\left(R\right)}=0.1$ curves in Fig. 3C and $\tau_{I}^{\text{decay}\left(R\right)}=8$-ms curves in Fig. 3E]. When E/I balance is spatially destabilized in the receiver network by increasing the breadth of recurrent inhibitory connections, $\sigma_{I}^{\left(R\right)}$, spatiotemporal patterns emerge in the receiver causing a further decrease in dimensionality when compared to that of the sender network (Fig. 3C). By contrast, when E/I balance is instead spatially destabilized in the sender by increasing $\sigma_{I}^{\left(S\right)}$, then in addition to decreasing within-sender dimensionality locally, the decrease is propagated to the receiver (Fig. 3D).

Different results are obtained if E/I balance is temporally destabilized. When the time constant of the inhibitory synapses, $\tau_{I}^{\text{decay}}$, is increased in the receiver network, there is not much change in the dimensionality of the receiver activity (Fig. 3E, right). This suggests that global measures of within-area shared fluctuations cannot detect a temporal destabilization in the receiver, since they yield similar results to the standard network with stable activity. However, if E/I balance is instead temporally destabilized in the sender by increasing $\tau_{I}^{\text{decay}\left(S\right)}$, a decrease in dimensionality is observed in the sender (Fig. 3F, left). This decrease in dimensionality is not propagated to the receiver: All curves overlap, no matter the extent of spatiotemporal patterns inherited from the sender (Fig. 3F, right). Hence, within-area dimensionality is differentially affected by temporal destabilization in the sender or receiver networks. We emphasize that in all scenarios, the sender is only given independent and identically distributed Poisson input. This may be the reason why it is sensitive to temporal destabilization, as the inputs have no specific temporal structure. On the other hand, the receiver is given temporally and spatially correlated spiking activity from the sender. Similar to the observed growth of correlated activity in feedforward networks (*23*), this structure may dampen the receiver network’s sensitivity to emergent shared fluctuations locally.

A critical point to remark is that the shared dimensionality in the receiver network is low, irrespective of whether spatiotemporal patterns emerge locally within the receiver network or are inherited from the sender network (compare the right panels of Fig. 3, C and Fig. 3D, as well as of Fig. 3, E and F). This raises an important dilemma for the interpretation of changes in the structure of population-wide shared fluctuations. Namely, that a change in the dimension of population activity can either be due to a shift of the internal dynamics within a network or be inherited from shifts in the dynamics of upstream areas. This ambiguity prompts us to next consider how the sender and receiver networks directly communicate their shared fluctuations.

### Between-area communication strength depends on the origin of shared fluctuations

To measure the interaction between sending and receiving networks, we use a recently developed measure of brain area–to–brain area communication based on reduced-rank regression (*17*). Briefly, the activity in the receiver network, *R*, is predicted from the activity in the sender network, *S*, through a linear model: $\hat{R}={\mathrm{SB}}_{\text{RRR}}$. The rank of the regression matrix *B*_RRR_ is constrained to be a low optimal value m^*^ (see the “Between-area communication” section). Prediction performance is given by the comparison between *R* and $\hat{R}$, quantifying the ability of the sender population activity in linearly predicting the receiver population activity through a low-dimensional communication subspace. Similar to the section above, we randomly sampled 50 neurons from a disc in both the sender and the receiver networks. The two discs are exactly overlapping to maximize communication strength (although the sampled neurons within the discs are not exactly overlapping because of random sampling). We then compute communication between the selected neurons in the sender and receiver.

**Fig. 4.**
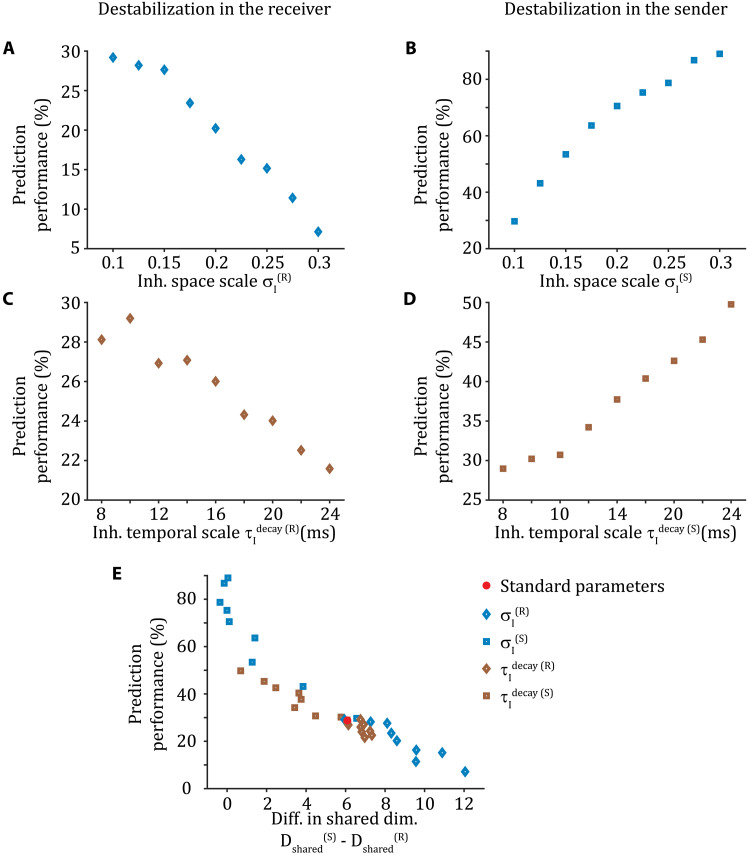
Between-area communication strength depends on the origin of shared fluctuations. (**A**) When E/I balance is spatially destabilized by broadening the inhibitory space scale σ_I_ in the receiver, prediction performance of the communication subspace decreases. (**B**) When E/I balance is spatially destabilized by increasing σ_I_ in the sender, prediction performance of the communication subspace increases. (**C** and **D**) Similar to (A) and (B), when E/I balance is temporally destabilized by increasing the inhibitory timescale $\tau_{I}^{\text{decay}}$. (**E**) Prediction performance of the communication subspace is higher when there is a good match in shared dimensionality between sender and receiver [small difference in $D_{\text{shared}}^{\left(S\right)}-D_{\text{shared}}^{\left(R\right)}$] than when the sender is much higher dimensional than the receiver [large difference in $D_{\text{shared}}^{\left(S\right)}-D_{\text{shared}}^{\left(R\right)}$]. Neurons are sampled from a disc with a radius of 0.2; symbols are centered in the mean and include the SEM.

When E/I balance is destabilized by increasing the breadth of recurrent inhibitory connections in the receiver, given by $\sigma_{I}^{\left(R\right)}$, the prediction performance of the communication subspace decreases (Fig. 4A). We note that the exact value of prediction performance depends on the spatial scale from which neurons are sampled: The larger the spatial scale, the lower the prediction performance is (fig. S4). By contrast, when E/I balance is instead spatially destabilized in the sender by increasing $\sigma_{I}^{\left(S\right)}$, the prediction performance of the communication subspace increases (Fig. 4B). If E/I balance is temporally destabilized by increasing the time constant of inhibitory neurons, $\tau_{I}^{\text{decay}}$, in either the sender or receiver networks, then qualitatively similar results are obtained (Fig. 4, C and D). Last, we observe that the optimal rank of the reduced-rank regression matrix *m*∗, which corresponds to the dimension of the communication subspace, decreases as the receiver E/I balance is more destabilized, while it increases as the sender E/I balance is destabilized (fig. S5). In effect, *m*∗ is mimicking prediction performance of the communication subspace (Fig. 4, A to D). In any case, the optimal dimension of the communication subspace is much lower than the theoretical upper bound of 50 (fig. S5).

In sum, despite similar dynamics in the receiver population regardless of the origin of E/I destabilization (Fig. 3, C to F), the prediction performance of the linear communication subspace is unambiguous to the origin: Receiver network destabilization disrupts communication, while sender network destabilization improves communication. To provide a framework to organize these disparate results, we compute the difference in the shared dimensionality in the sender and receiver networks: $D_{\text{shared}}^{\left(S\right)}-D_{\text{shared}}^{\left(R\right)}$. Notably, this difference determines the prediction performance across these varied datasets, irrespective of whether E/I balance is spatially or temporally destabilized in either the sender or the receiver (Fig. 4E). E/I destabilization in the sender through either broad or slow inhibition (Fig. 4E, blue and brown squares, respectively) increases prediction performance compared to the standard network (Fig. 4E, red disc). By contrast, the same destabilization in the receiver decreases the prediction performance compared to the standard network (Fig. 4E, blue and brown diamonds, respectively).

Last, we note that a match in within-area shared dimensionality in the sender and receiver promotes faithful linear communication but this is not a sufficient condition. We show that if there is a misalignment of shared variability in the sender and receiver, for example, through emergence of different types of spatiotemporal patterns in the sender and receiver, then linear communication is disrupted (fig. S6).

### Linear communication can be disrupted in two general ways

To summarize the results above, we promote faithful communication as assessed by a linear measure if shared dimensionality is similarly low in the sender and receiver networks. If the sender network displays high-dimensional shared variability while the receiver network exhibits low-dimensional shared variability, such as when spatiotemporal patterns of activity emerge in the receiver network, then communication is disrupted (Figs. 3, C and E, and 4, A and C). We now investigate the underlying mechanisms by which linear communication between the sender and receiver networks can be disrupted.

**Fig. 5.**
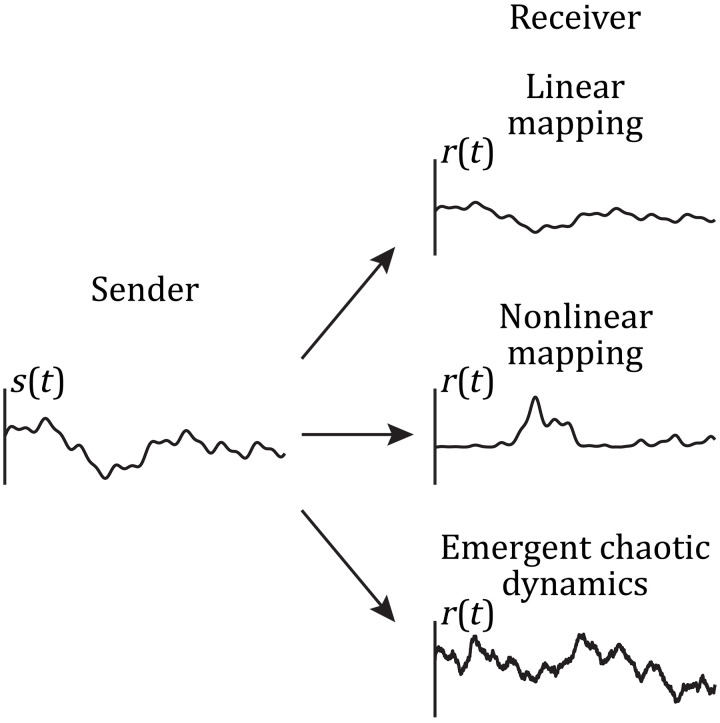
Linear communication can be disrupted in two general ways. For illustration purposes, we represent the average activity in the sender and receiver as a scalar time series, *s*(*t*) and *r*(*t*), respectively. We set *s*(*t*) as a sum of six sine functions with amplitudes ranging over [0.05, 0.3], frequencies over [0.5, 10], and phases over [0, 2π]. If the mapping between sender and receiver is linear, such as the compression *r*(*t*) = 0.5*s*(*t*) (top receiver), then linear measures are suitable to uncover between-area communication. Rather, if the activity in the receiver is obtained through a nonlinear mapping of the activity in the sender, such as *r*(*t*) = −0.8*s*(*t*)[*s*(*t*) − 0.5][*s*(*t*) − 1] (middle receiver), then linear communication appears disrupted, although the receiver is effectively driven by the sender. Last, if dynamic fluctuations emerge in the receiver, which are uncorrelated with the sender activity, then these fluctuations would act as noise and degrade the linear communication between sender and receiver. In this illustration, we model these fluctuations as an additive Ornstein-Uhlenbeck process *n*(*t*) with τ = 10 s and σ = 0.05 and set *r*(*t*) = *s*(*t*) + *n*(*t*) (bottom receiver).

If the within-area dimensionality of the sender and receiver networks is matched and their interaction is linear, then their communication is faithful (Fig. 5, top case). We outline two main hypothesized mechanisms by which linear communication can be disrupted. On one hand, because communication is assessed by a linear measure, if the mapping between sender and receiver activities is highly nonlinear, then communication will appear disrupted (Fig. 5, middle case). However, in this case, the sender does effectively drive the receiver, yet this communication is blind to any linear metric. On the other hand, while the mapping of activity from sender to receiver could be linear, emergent dynamics in the receiver that is unrelated to the sender network activity may hinder communication (Fig. 5, bottom case). In this case, the emergent activity appears as “noise” and corrupts the relation between the sender and receiver networks. In the remaining sections, we show that the results presented above are classified into one of these two generic categories.

### Spatial but not temporal destabilization of E/I balance downstream yields a nonlinear mapping from the sender to the receiver

As mentioned above, we start by determining whether the mapping of neuronal activity from the sender to receiver networks remains linear despite destabilization of E/I balance in the receiver. We use a standard approach to investigating linearity. Basically, two scenarios are compared. If the sum of the receiver activity (also known as output) to two different inputs is identical to the receiver activity in response to the sum of the two inputs, then it indicates that the sender-receiver mapping is linear.

**Fig. 6.**
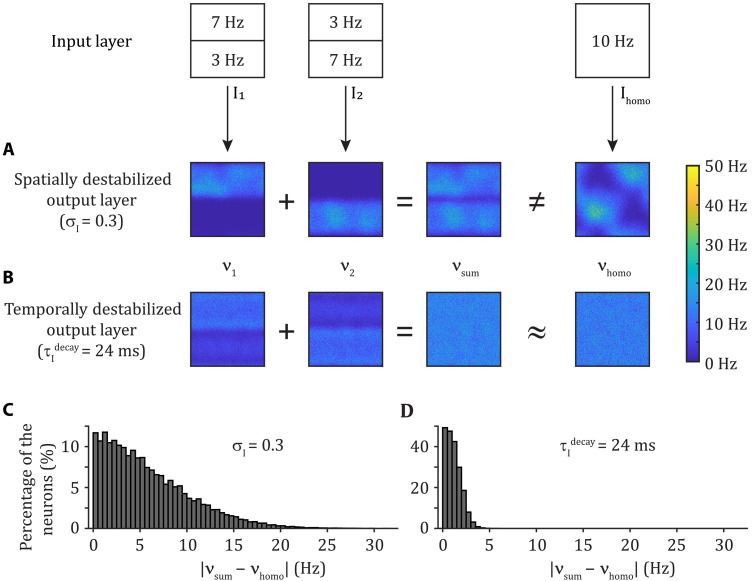
Spatial but not temporal destabilization of E/I balance downstream yields a nonlinear mapping from the sender to the receiver. The firing rate of the Poisson processes in the input layer is set to 7 Hz for the upper input neurons and 3 Hz for the lower neurons (*I*_1_), or the opposite (*I*_2_), or at 10 Hz for all input neurons (*I*_homo_). We compute the output layer response as ν_1_, ν_2_, and ν_homo_ to these respective cases and define ν_sum_ = ν_1_ + ν_2_. (**A**) When the output layer is spatially destabilized (σ_I_ = 0.3), we have that ν_sum_ ≠ ν_homo_. (**B**) When the output layer is temporally destabilized ($\tau_{I}^{\text{decay}}=24$ ms), we have that ν_sum_ ≈ ν_homo_. (**C** and **D**) Distribution of the absolute difference in firing rates between ν_sum_ and ν_homo_ for the case with a spatially destabilized (C) or a temporally destabilized (D) output layer. Each trial lasts 20 s, from which we remove the first 1 s and estimate the firing rate of each neuron. The panels show the results for one trial. Over 30 trials, the mean absolute difference in firing rate is (μ ± SD): 5.3 ± 4.2 Hz for the spatially destabilized network and 1.1 ± 0.8 Hz for the temporally destabilized network.

Specifically, we consider the activity of a destabilized output (receiver) network in response to an input (sender) population modeled by temporally homogeneous, independent Poisson spike trains. We compare the activity of the recurrently coupled output network in response to two scenarios (Fig. 6). In the first scenario, spatially heterogeneous inputs are used. Input *I*_1_ has average firing rates of 7 Hz in the upper half and average firing rates of 3 Hz in the lower half of the spatial grid. We record the (vectored) output neuron firing rates ν_1_ in response to *I*_1_. We repeat this experiment with input *I*_2_, where the upper and lower half average firing rates are switched, and we record response rates as ν_2_. We construct the response to the summed input *I*_1_ + *I*_2_ to be ν_sum_ = ν_1_ + ν_2_. In the second scenario, spatially homogeneous input *I*_homo_ = *I*_1_ + *I*_2_ is used. Thus, the average firing rate is 10 Hz in the whole spatial grid. We record the response to this sum of inputs as ν_homo_. A linear mapping between input and output would predict that ν_sum_ = ν_homo_.

A spatial destabilization of E/I balance in the output layer shows a summed response activity that is different from the activity in response to the homogeneous input (Fig. 6A). The lateral inhibition in the output layer shapes the response to the homogeneous input, which disagrees with a simple linear mixing of the responses. Beyond a simple visual inspection of the spatial distribution of firing rates, we compute for every neuron *j* in the output network the comparison ∣ν_sum,*j*_−ν_homo,*j*_∣ (Fig. 6C). The absolute difference is large, indicating a complete reshuffling of individual neuron firing rates between scenarios. This implies that across the output network, we have ν_sum_ ≠ ν_homo_. Thus, a spatially destabilized E/I balance imparts a clear nonlinear mapping between input and output spiking activities.

By contrast, a temporally destabilized E/I balance in the output network produces no obvious change in spatial distribution of firing rates (Fig. 6B). When individual neurons are compared across scenarios, we observe only a slight change between ν_sum,*j*_ and ν_homo,*j*_ (Fig. 6D), so that we have across the output network ν_sum_ ≈ ν_homo_. Hence, a linear mapping of activity is qualitatively maintained despite temporal destabilization of the output layer.

These results suggest that when the receiver E/I balance is spatially destabilized, a nonlinear mapping between the sender and receiver activities contributes to the disruption of linearly measured communication (Figs. 4A and 6, A and C). However, while temporal destabilization in the receiver disrupts linear communication (Fig. 4C), it does maintain a sender to receiver linear mapping (Fig. 6, B and D). In the next section, we explore the hypothesis that temporal destabilization induces emergent dynamics in the receiver network, which corrupt the communication between the sender and receiver.

### Temporal but not spatial destabilization of E/I balance yields macroscopic emergent dynamics in the receiver

**Fig. 7.**
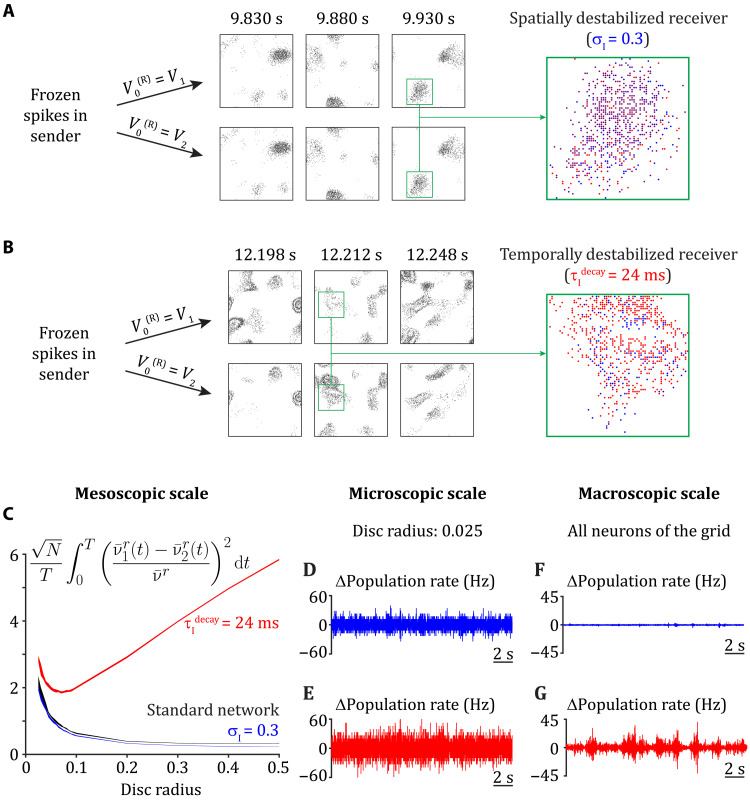
Temporal but not spatial destabilization of E/I balance yields macroscopic emergent dynamics in the receiver. (**A** and **B**) A single realization of spiking activity is generated in the input layer and propagated to the sender. The membrane potential of neuron *j* in the receiver is set to one of two different initial conditions: $V_{j}^{1}$ or $V_{j}^{2}$ (randomly generated for each neuron). Spike time raster plots (Δ*t* = 2 ms) at three time points are presented for the networks with $\sigma_{I}^{\left(R\right)}=0.3$ (A) and $\tau_{I}^{\text{decay}\left(R\right)}=24$ ms (B). Raster plot zoom in: Blue dots are spike times for the first trial, red dots are spike times for the second trial, and purple dots are overlapping spike times of the first and second trials. (**C**) Normalized difference in population activity for neurons sampled from a disc of radius *r*, with *N* as the number of neurons within the disc, *T* as the length of the trials (20 s), ${\bar{\nu }}_{\alpha }^{r}\left(t\right)$ for α ∈ {1,2} as the average firing rate over all neurons of the disc for realizations 1 and 2, and ${\bar{\nu }}^{r}$ as the average firing rate over all neurons within the disc from both trials and all time points. (**D** and **E**) Difference in the average firing rate over all neurons sampled from a random disc with a radius of 0.025 (96 neurons) between the two trials as a function of time: for the spatially destabilized case (D) and the temporally destabilized case (E). (**F** and **G**) Same as (D) and (E), but for the average firing rate over all excitatory neurons on the grid (40,000).

Destabilization of E/I balance in the receiver network, through either broad or slow inhibition, produces rich, spatiotemporal patterns of spiking activity (Fig. 2). However, it is unclear whether this activity corrupts communication between the sender and receiver networks. To determine this, we generate a single (frozen) realization of input spike trains from the input layer and record the resulting spike train activity from the sender layer. To this frozen sender input, we compare two response trials in the receiving layer, where the only difference between them is the initial membrane voltage of the excitatory neurons in the receiving network (Fig. 7, A and B). If the patterns of spiking activity in the receiver network differ significantly between trials despite the sender activity being frozen, then this would indicate an emergent, chaotic dynamic owing to complex recurrent interactions (*48*–*50*). In this case, the differing receiver network responses across trials would act as noise that corrupts the communication between the sender and receiver.

When E/I balance in the receiver network is spatially destabilized, the difference of initial membrane potential conditions does not affect the overall macroscopic (aggregated) patterns of spiking activity across the two trials (Fig. 7A). By contrast, when the receiver is temporally destabilized, we observe notable discrepancy in the macroscopic spatiotemporal patterns between the two trials (Fig. 7B). These initial results support our hypothesis that temporal E/I destabilization induces an emergence of chaotic dynamics at the population-wide scale, which is the source of linear communication disruption. However, even during spatial E/I destabilization, while the macroscopic (population-wide) network activity is reliable across trials, the microscopic activity is nevertheless unreliable. The exact spike train sequences across the network differ substantially between the two trials (Fig. 7A, zoom in), so that any observer of a single neuron would easily distinguish the trials. These results agree with previous studies where a weak perturbation to network activity yields only a transient change in firing rate but a long-lasting trial-to-trial decorrelation of spike sequences in spatially disordered balanced networks (*49*, *50*). The differential spatial scale (micro versus macro) of unreliability in spatially and temporally E/I destabilized networks prompts a more detailed analysis.

We consider the trial-to-trial reliability of the spiking activity of neurons aggregated over a spatial disc of radius *r*. Across a trial, we sum the instantaneous spiking activity of the neurons within the disc, ν*^r^*(*t*). We integrate the normalized difference of ν*^r^*(*t*) between the two trials as a measure of unreliability (Fig. 7C). Spatial destabilization only yields microscopic unreliability, as observed for discs of small radii [Fig. 7, C (blue curve), D, and F], similar to the standard network in the stable regime (Fig. 7C, compare blue and black curves). In contrast, a temporal destabilization induces macroscopic unreliability in addition to microscopic unreliability [Fig. 7, C (red curve), E, and G). These results extend our understanding of rich spiking neuronal dynamics in networks with biologically constrained architecture and are consistent with the observation of macroscopic chaotic dynamics upon temporal, but not spatial, destabilization of E/I balance in rate networks (*51*).

## DISCUSSION

The mechanisms that produce low-dimensional shared variability across a neuronal population can be organized into two broad categories. First, shared variability of the neurons in a brain region may be inherited (in part) from connecting brain areas (*17*, *52*, *53*). Second, shared variability may be an emergent property of a brain area, owing to local, complex recurrent interactions between neurons (*28*–*30*, *32*). Population recordings restricted to a single brain area cannot easily disentangle the contributions of each mechanic to the total shared variability. Rather, multiarea brain recordings will be needed to expose these separate mechanisms (*1*). In our study, we explored signatures of inherited or emergent shared variability within a brain area by measuring the (linear) communication between distinct, but connected, brain areas.

A suitable modeling framework to investigate the interplay between inheritance and emergence of neuronal variability in brain circuits has only recently become available. At one extreme, the inheritance of neuronal fluctuations has been extensively studied through the analysis of activity propagation in feedforward networks (*12*, *20*, *21*, *23*). However, since those circuits explicitly lacked within layer recurrent connections, they could not model the emergence of complex population dynamics. At the other extreme, within-population recurrent, yet unstructured, excitatory and inhibitory connections were introduced to model asynchronous activity reminiscent of the baseline state of cortical activity (*24*–*26*). However, these networks did not produce rich, low-dimensional fluctuations shared across the population. Over the past few years modeling frameworks have included structure to the within-population recurrent wiring that permits low-dimensional fluctuations to intrinsically emerge within the network. Different approaches have been taken. On one hand, forcing a low-rank structure of the recurrent connectivity matrix yields low-dimensional activity, revealing a strong relationship between structure and dynamics (*29*, *30*). On the other hand, low-dimensional within-area shared fluctuations can emerge because of a destabilization of E/I balance despite a connectivity matrix with high rank (*27*, *28*, *32*). In our work, we leverage those last modeling frameworks to investigate how the emergence and inheritance of low-dimensional neuronal fluctuations affect between-area interactions in well-controlled settings.

We assess the interaction between a sender network and a receiver network through a linear communication measure. Brain recordings are believed to frequently operate in a linear regime, particularly in early sensory areas (*54*). Besides, linear methods are routinely used for the analysis of neuronal dynamics and, in many cases, have proven successful. At the single-population level, they have unraveled stable low-dimensional manifolds in working memory networks (*3*) and in motor areas (*5*). In addition, the suppression of rich spatiotemporal dynamics has been shown to increase the amount of linear Fisher information that is propagated down successive layers (*55*). Nonetheless, decision-making is now thought to arise through the interaction of distributed brain regions (*1*). Recent technological advances have allowed a glimpse into distributed processing through simultaneous recordings from distinct neuronal populations, allowing us to answer the question of how much of the dynamics in a population can be explained by the activity of another recorded brain area (*8*, *9*). At this multiple-population level, linear methods have also been critical. They have exhibited selectivity (*46*) and a low-dimensional subspace (*17*, *18*, *56*) in the communication between brain areas. Our work shows that those methods provide a useful lens to investigate the inheritance of neuronal fluctuations by a receiver displaying linear mapping, particularly in low-dimensional inheritance settings.

However, when the receiving area is in a nonlinear regime where complex spatiotemporal dynamics intrinsically emerge, we show that between-area communication cannot be properly assessed by linear measures. Our results support a recent study revealing that when a network exhibits strong pairwise correlations, reminiscent of low-dimensional pattern formation, connectivity inference is biased toward an excess of connectivity between highly correlated neurons (*15*). Linear inference methods are only appropriate when neuronal dynamics operate in a linear regime, where activity is high-dimensional and unstructured (*15*). Although early sensory areas are believed to mostly operate in a linear regime to faithfully encode sensory inputs, brain areas involved in higher-level cognition exhibit low-dimensional spatiotemporal dynamics (*45*, *56*–*59*). Further, it has recently been shown that microscopic irregularity can subsist even in the presence of macroscopic fluctuations (*28*, *60*). Our results indicate that even when the receiver network is in the pattern-forming regime due to a spatial destabilization, it is nevertheless reliably driven by the sender network, as reflected by a lack of macroscopic chaos. However, a nonlinearity in the sender-receiver mapping makes linear methods of communication blind to this interaction. Alternatively, emergence of unreliable macroscopic spiking dynamics due to temporal destabilization also impair linear measures of communication although the interaction is effectively linear. Therefore, novel nonlinear methods, as well as ways to characterize emergent fluctuations, will have to be developed to accurately assess communication involving brain areas with more complex dynamics, such as those involved in higher-level brain regions associated with cognition.

Note that our networks are formally chaotic for all the parameter choices we have studied. It is well known that networks of spiking neuron models with strong synaptic coupling show unpredictable dynamics with an extreme sensitivity to the presence or absence of an individual spike (*49*, *50*). Because of this, in our study, both sender and receiver networks are always in a chaotic regime. In this work, however, we make a distinction between the spatial scales of unpredictable dynamics, with chaos restricted to small groups being termed microscopic, while population-wide unreliability is termed macroscopic chaos. We observe that when only microscopic chaos is present, interaction between areas can be well assessed by linear methods, consistent with (*56*). When chaotic dynamics are observable at the macroscopic scale, then linear methods are ineffective in measuring communication between areas.

Feedforward networks have a long history. They have been used to investigate neuronal activity propagation, synchronization, and information transmission (*12*, *20*, *21*, *23*, *55*). Recently, they have extensively been used to model the primate visual system and in machine learning applications such as object recognition (*19*). Despite their simplicity relative to highly recurrent biological networks, they have already brought tremendous value to the community. In this work, we take advantage of this simplified modeling framework to thoroughly study the interplay between inheritance and emergence of neuronal fluctuations in tightly controlled settings. The use of feedforwardly connected distinct sender and receiver networks, each only involving local recurrent connections, allows us to observe the differential effects of low-dimensional within-area shared fluctuations on between-area communication depending on their well-defined origin. However, brain circuits show recurrent architecture spanning a wide range of spatial scales. Furthermore, cognition is believed to arise from distributed computational processes. Even in early sensory areas historically thought of as mostly feedforward, the importance of feedback interactions in sensory processing has started to be exposed (*61*). Therefore, a natural extension of our work will be the implementation of more complex circuit architectures, starting by the introduction of feedback connections, to provide novel insights into the mechanistic interplay of inheritance and emergence of within-area shared fluctuations across spatial scales.

## MATERIALS AND METHODS

### Network structure

A three-layer spiking network model is implemented similar to the previous work (*32*). The input layer consists of 2500 excitatory neurons whose spikes are taken from independent homogeneous (space and time) Poisson processes with a uniform rate of 10 Hz. The sender and receiver layers each consist of 40,000 excitatory (E) and 10,000 inhibitory (I) neurons that are arranged on a unit square domain Γ = [0,1] × [0,1] with periodic boundary conditions. The probability of connection between a presynaptic neuron belonging to class β ∈ {E, I} located at position $\vec{y}=\left(y_{1},y_{2}\right)$ and a postsynaptic neuron belonging to class α ∈ {E, I} located at position $\vec{x}=\left(x_{1},x_{2}\right)$ depends on their pairwise distance measured periodically on Γ

$$p_{\alpha \beta }\left(\vec{x},\vec{y}\right)=\frac{K_{\alpha \beta }^{\text{out}}}{N_{\alpha }}g\left(x_{1}-y_{1},\sigma_{\beta }\right)g\left(x_{2}-y_{2},\sigma_{\beta }\right)$$ (1)

where $K_{\alpha \beta }^{\text{out}}$ is the out-degree, so ${\bar{p}}_{\alpha \beta }=\frac{K_{\alpha \beta }^{\text{out}}}{N_{\alpha }}$ is the mean connection probability, and *g*(*u*, σ) is a wrapped Gaussian distribution

$$g\left(u,\sigma \right)=\frac{1}{\sigma \sqrt{2\pi }}\sum_{k=-\infty }^{+\infty }e^{{\left(-u+k\right)}^{2}/\left(2\sigma^{2}\right)}$$ (2)

Excitatory feedforward connections between layers and recurrent excitatory and inhibitory connections within layers are spatially distributed according to a Gaussian with width σ_ffwd_, σ_E_, and σ_I_, respectively.

### Neuronal dynamics

Excitatory and inhibitory neurons in the sender and receiver networks are modeled as conductance-based exponential integrate-and-fire neurons

$$C_{m}\frac{dV_{j}^{\alpha }}{dt}=-g_{L}^{\alpha }\left(V_{j}^{\alpha }-V_{L}\right)+g_{L}^{\alpha }\Delta_{T}^{\alpha }e^{\left(V_{j}^{\alpha }-V_{T}\right)/\Delta_{T}^{\alpha }}+I_{j}^{\alpha }$$ (3)

with α ∈ {E, I}. A neuron spikes when its membrane voltage $V_{j}^{\alpha }$ reaches the spiking threshold *V*_th_. Then, its membrane voltage is reset at *V*_reset_ for a refractory period $t_{\text{ref}}^{\alpha }$. The total current received by neuron *j* belonging to class α, $I_{j}^{\alpha }\left(t\right)$, is given by the summation of feedforward and recurrent input

$$\begin{array}{c} \frac{I_{j}^{\alpha }\left(t\right)}{C_{m}}=\sum_{k=1}^{N_{E}^{\text{ffwd}}}\frac{j_{\mathrm{jk}}^{\text{ffwd}}}{\sqrt{N}}\sum_{n}\eta_{E}\left(t-t_{n}^{k}\right)\\ +\sum_{\beta \in \left\{E,I\right\}}\sum_{k=1}^{N_{\beta }^{\text{rec}}}\frac{j_{\mathrm{jk}}^{\text{rec}}}{\sqrt{N}}\sum_{n}\eta_{\beta }\left(t-t_{n}^{k}\right) \end{array}$$ (4)

with *N* = *N*_E_ + *N*_I_ as the total number of neurons within the layer of interest. The postsynaptic current, η_β_(*t*), is induced by presynaptic spiking and depends on the class (β ∈ {E, I}) of the presynaptic neuron. Assuming a single presynaptic spike at time *t* = 0, it is given by the difference of two exponentials with rise timescale $\tau_{\beta }^{\text{rise}}$ and decay timescale $\tau_{\beta }^{\text{decay}}$

$$\eta_{\beta }\left(t\right)=\left\{\begin{array}{cc} \frac{e^{-t/\tau_{\beta }^{\text{decay}}}-e^{-t/\tau_{\beta }^{\text{rise}}}}{\tau_{\beta }^{\text{decay}}-\tau_{\beta }^{\text{rise}}}, & t⩾0\\ 0, & t<0 \end{array}\right.$$ (5)

Equations are numerically integrated using the forward Euler method with a timestep of 0.05 ms. Instantaneous firing rates were computed from the spike counts in nonoverlapping 50-ms bins. Unless specified otherwise, all neuronal and connectivity parameters are identical in the sender and the receiver layers (Table 1).

### Factor analysis

Shared covariance of within-area neuronal activity is assessed through FA (*41*, *42*)

$$p\left(\vec{x}\right)=N\left(\vec{\mu },C_{\text{shared}}+C_{\text{private}}\right)$$ (6)

where $\vec{x}$ is a vector of spike counts across the selected neurons and $\vec{\mu }$ is a vector of mean spike counts. The shared component of the full covariance matrix is *C*_shared_ = *LL^T^*, where *L* is the loading matrix relating the latent variables to the neural activity. The private component of the full covariance matrix is *C*_private_. It is a diagonal matrix whose elements represent the individual neuronal variances. The expectation-maximization algorithm was used to estimate the model parameters $\vec{\mu }$, *L*, and *C*_private_. To determine the number of latent variables and, consequently, the rank of *C*_shared_, the cross-validated data likelihood was maximized. We use the publicly available code (https://users.ece.cmu.edu/~byronyu/software.shtml) for the implementation of FA (https://github.com/ogozel/fa_Yu).

### Within-area shared dimensionality

Singular value decomposition is applied to *C*_shared_

$$C_{\text{shared}}=U\Lambda U^{T}$$ (7)

where the columns of *U* are the eigenvectors and the elements of the diagonal matrix Λ, λ*_i_*, are the associated eigenvalues ordered from larger to smaller.

The dimensionality of the shared covariance matrix is estimated using the effective dimension (sometimes referred to as the participation ratio) (*43*, *44*)

$$D_{\text{shared}}=\frac{{\left(\sum_{i=1}^{N}\lambda_{i}\right)}^{2}}{\sum_{i=1}^{N}\lambda_{i}^{2}}$$ (8)

### Between-area communication

To assess communication between sender and receiver networks, we use a recently developed communication subspace measure based on reduced-rank regression (*17*)

$$\hat{R}={\mathrm{SB}}_{\text{RRR}},\;\text{with}\;\text{rank}\left(B_{\text{RRR}}\right)=m$$ (9)

with *S* as the activity in the sender network and $\hat{R}$ as the estimated activity in the receiver network (both of size *T* × *K*]). *T* is the total number of time points, and *K* is the number of sampled neurons (we set *K* = 50 in both the sender and the receiver). The reduced-rank regression matrix, *B*_RRR_, is a low-rank approximation of the ordinary least-squares solution (*17*)

$$B_{\text{RRR}}=B_{\text{OLS}}\Gamma_{m}\Gamma_{m}^{T}$$ (10)

with *B*_OLS_ = (*S^T^S*)^−1^*S^T^R* as a full rank regression matrix. Hence, Γ*_m_* is a matrix whose columns are the *m* first eigenvectors of the covariance matrix of the receiver activity estimated using the ordinary least-squares solution: (*SB*_OLS_)*^T^*(*SB*_OLS_) = ΓΛΓ*^T^*. The dimensions in activity *S* that are most predictive of activity *R* according to the communication subspace measure are called “predictive dimensions.” They are the *m* columns of *B*_OLS_Γ*_m_*.

We define the prediction performance as the *R*^2^ value [residual sum of squares (RSS), total sum of demeaned squares (TSS)]

$$1-\frac{\text{RSS}}{\text{TSS}}=1-\frac{\sum_{k=1}^{K}\sum_{t=1}^{T}{\left(R_{k}^{t}-{\hat{R}}_{k}^{t}\right)}^{2}}{\sum_{k=1}^{K}\sum_{t=1}^{T}{\left(R_{k}^{t}-<R_{k}{>}_{T}\right)}^{2}}\in \left[0,1\right]$$ (11)

where $<R_{k}{>}_{T}=\frac{1}{T}\sum_{t=1}^{T}R_{k}$. In the results, we multiply the prediction performance by 100 to get percentages.

For each network parameter set, we compute prediction performance of a communication subspace with rank *m*, *m* ∈ {0,1, …, *K*}. We define the optimal rank *m*∗ as the smallest number of dimensions for which the prediction performance is within one SEM of the peak performance over 20 cross-validation folds. In the results, we report the average prediction performance using *B*_RRR_ with optimal rank *m*∗ over 20 cross-validation folds.

## Supplementary Materials

### This PDF file includes:

Supplementary Methods Figs. S1 to S7 References
