# Supplementary material for: Between-area communication through the lens of within-area neuronal dynamics
Source: Sci Adv. 2024 Oct 16;10(42):eadl6120. doi: 10.1126/sciadv.adl6120 (PMC11482330; doi:10.1126/sciadv.adl6120)
Supplement: Supplementary file 1 — Supplementary Methods Figs. S1 to S7 References [file sciadv.adl6120_sm.pdf]

Science Advances
AAAS

Supplementary Materials for

Between-area communication through the lens of within-area neuronal dynamics

Olivia Gozel and Brent Doiron

Corresponding author: Olivia Gozel, gozel@uchicago.edu; Brent Doiron, bdoiron@uchicago.edu

Sci. Adv. **10**, eadl6120 (2024)
DOI: 10.1126/sciadv.adl6120

This PDF file includes:

Supplementary Methods
Figs. S1 to S7
References

Supplementary Methods

Alignment of shared variability

We hypothesize that a misalignment of the low dimensional manifolds of shared variability in the sender and receiver networks is the cause of disruption of their communication. To measure the alignment of shared variability in sender and receiver networks, we use a method based on canonical correlation analysis which has recently been used to align within-area latent dynamics over several days where a different number of neurons with different identities were recorded (*62*). We know from equation (7) that the singular value decomposition of the shared covariance matrix is given by: $C_{\text{shared}} = U\Lambda U^T$. Hence the columns of U are the eigenvectors of C_{shared}, ordered from the one which explains the most shared variance to the one that explains the least. We keep $m = 20$ shared latent variables, as they are sufficient to explain more than 90% of the shared variance. Therefore, we only keep the first m columns of U: U_m of size $[K \times m]$ with $K = 50$ the number of sampled neurons. For the sender and receiver networks separately, we project the activity of the K sampled neurons onto the m shared latent variables: $L_k = XU_m$. We thus obtain two $[T \times m]$ matrices of latent dynamics L_k, where T is the number of timepoints, $k \in \{S, R\}$ and X is the corresponding activity in the sender (S), or in the receiver (R), of size $[T \times K]$. Then we compute the QR decomposition of the shared latent dynamics: $L_k = Q_k R_k$. The singular value decomposition of the covariance of Q_S with Q_R is given by: $Q_S^T Q_R = \tilde{U}\tilde{S}\tilde{V}^T$. Canonical correlation analysis finds new latent directions to maximize the pairwise correlations between the sending and receiving populations. The projection of the shared latent dynamics onto these new latent directions is given by: $\tilde{L}_k = L_k M_k$, where $M_S = R_S^{-1}\tilde{U}$ and $M_R = R_R^{-1}\tilde{V}$. Finally, the aligned canonical correlations are given by the pairwise correlations between the aligned shared latent dynamics: $\tilde{L}_S^T \tilde{L}_R = \tilde{U}^T Q_S^T Q_R \tilde{V} = \tilde{S}$. They are the elements of the diagonal matrix $\tilde{S}$, which are ordered from larger to lower value. In our case, the value of the canonical correlation for each eigenmode indicates to what extent the given latent dimension can be well aligned between the sender and receiver networks through a linear transformation.

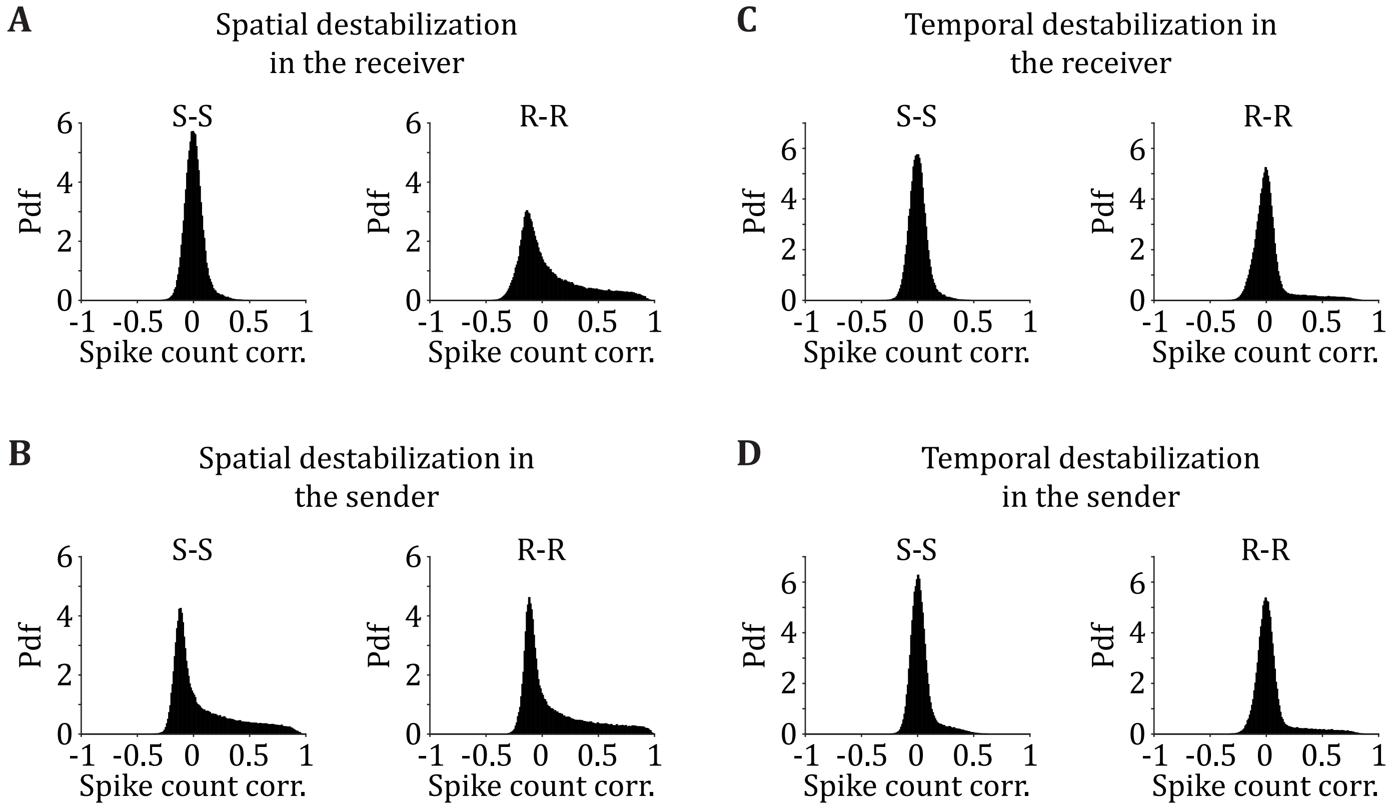

Figure S1: Within-area spike count correlations are differentially affected by the type of E/I balance destabilization.
(**A**) Probability density functions (pdfs) of the pairwise spike count correlations for neuron pairs within the sender area, S (left, mean of the distribution: $\mu = 0.0045$), and within the receiver area, R (right, $\mu = 0.0658$) when the E/I balance is destabilized spatially in the receiver ($\sigma_I^{(\mathrm{R})} = 0.3$). (**B**) Pdfs of the pairwise spike count correlations for neuron pairs within-area S ($\mu = 0.0839$), and within-area R ($\mu = 0.0866$) when the E/I balance is destabilized spatially in the sender ($\sigma_I^{(\mathrm{S})} = 0.3$). (**C**) Pdfs of the pairwise spike count correlations for neuron pairs within S ($\mu = 0.0043$), and within R (right, $\mu = 0.0237$) when the E/I balance is destabilized temporally in the receiver ($\tau_I^{\mathrm{decay\ (R)}} = 24$ ms). (**D**) Pdfs of the pairwise spike count correlations for neuron pairs within-area S ($\mu = 0.0269$), and within-area R ($\mu = 0.0394$) when the E/I balance is destabilized temporally in the sender ($\tau_I^{\mathrm{decay\ (S)}} = 24$ ms).

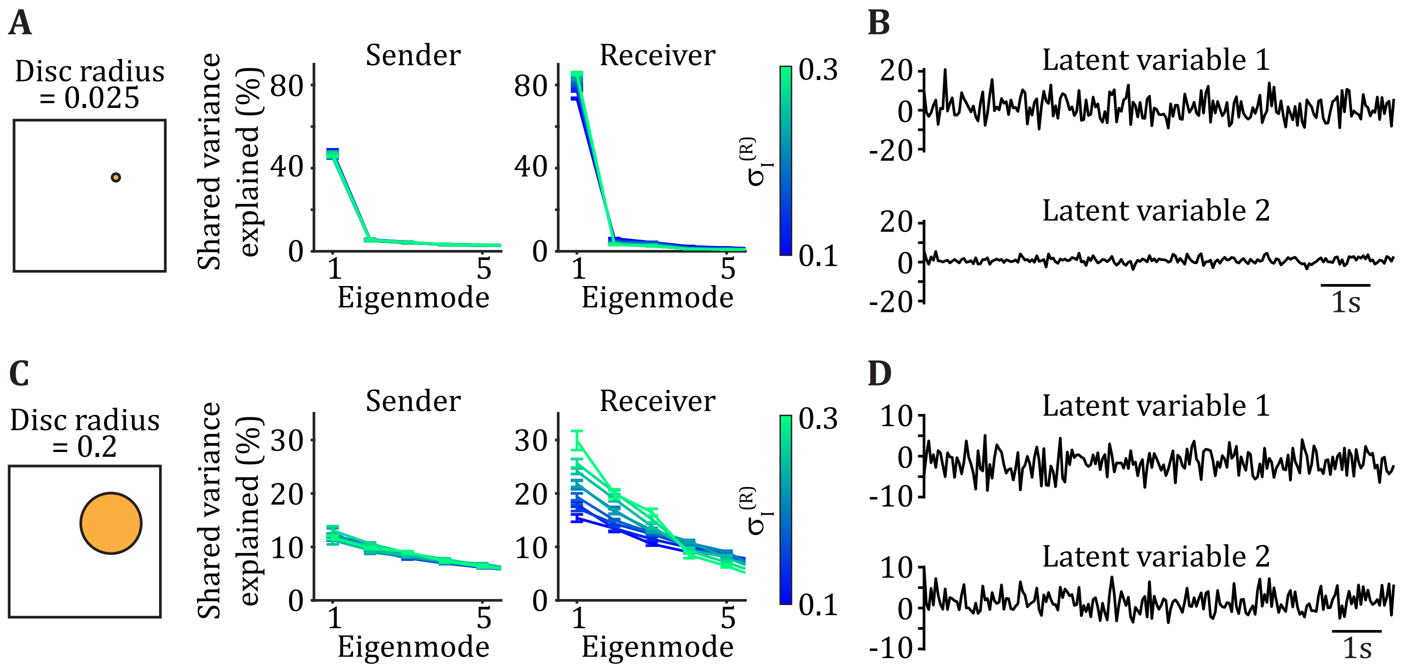

Figure S2: The structure of within-area shared fluctuations is spatial-scale dependent. (**A**) Shared variance explained by the first five eigenmodes within the sender network (S) and the receiver network (R) when modifying σ_I in R. Neurons are randomly sampled from a small disc (disc radius = 0.025). (**B**) Projection of 10 s of the receiving population activity on the first two shared eigenvectors to obtain the first two shared latent variables. Neurons are sampled from a small disc in the standard network ($\sigma_I^{(S)} = \sigma_I^{(R)} = 0.1$). (**C,D**) Same as (A,B), except that neurons are sampled from a large disc (disc radius = 0.2).

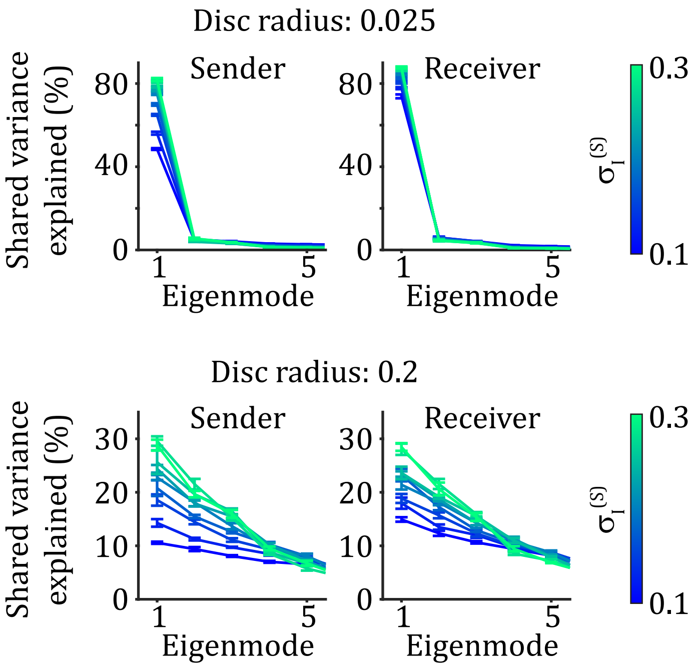

Figure S3: The structure of within-area shared fluctuations is spatial-scale dependent. Shared variance explained by the first five eigenmodes within sender network (S, left) and within receiver network (R, right) when modifying σ_I in S. Neurons are randomly sampled from a small disc (top) or from a large disc (bottom).

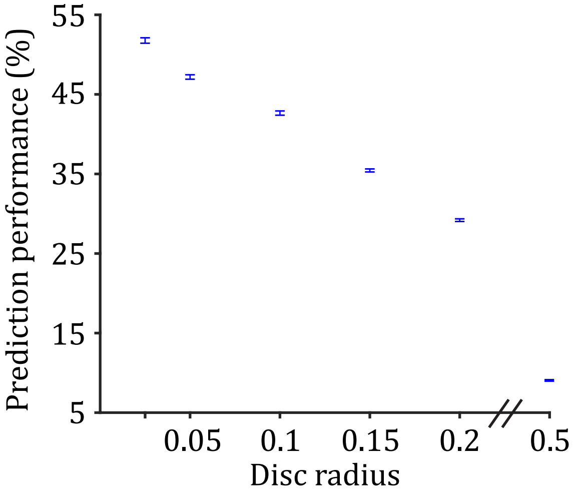

Figure S4: Dependence on the size of the spatial domain from which neurons are sampled on prediction performance of the communication subspace.
Scenario with the standard network parameters ($\sigma_I^{(S)} = \sigma_I^{(R)} = 0.1$).

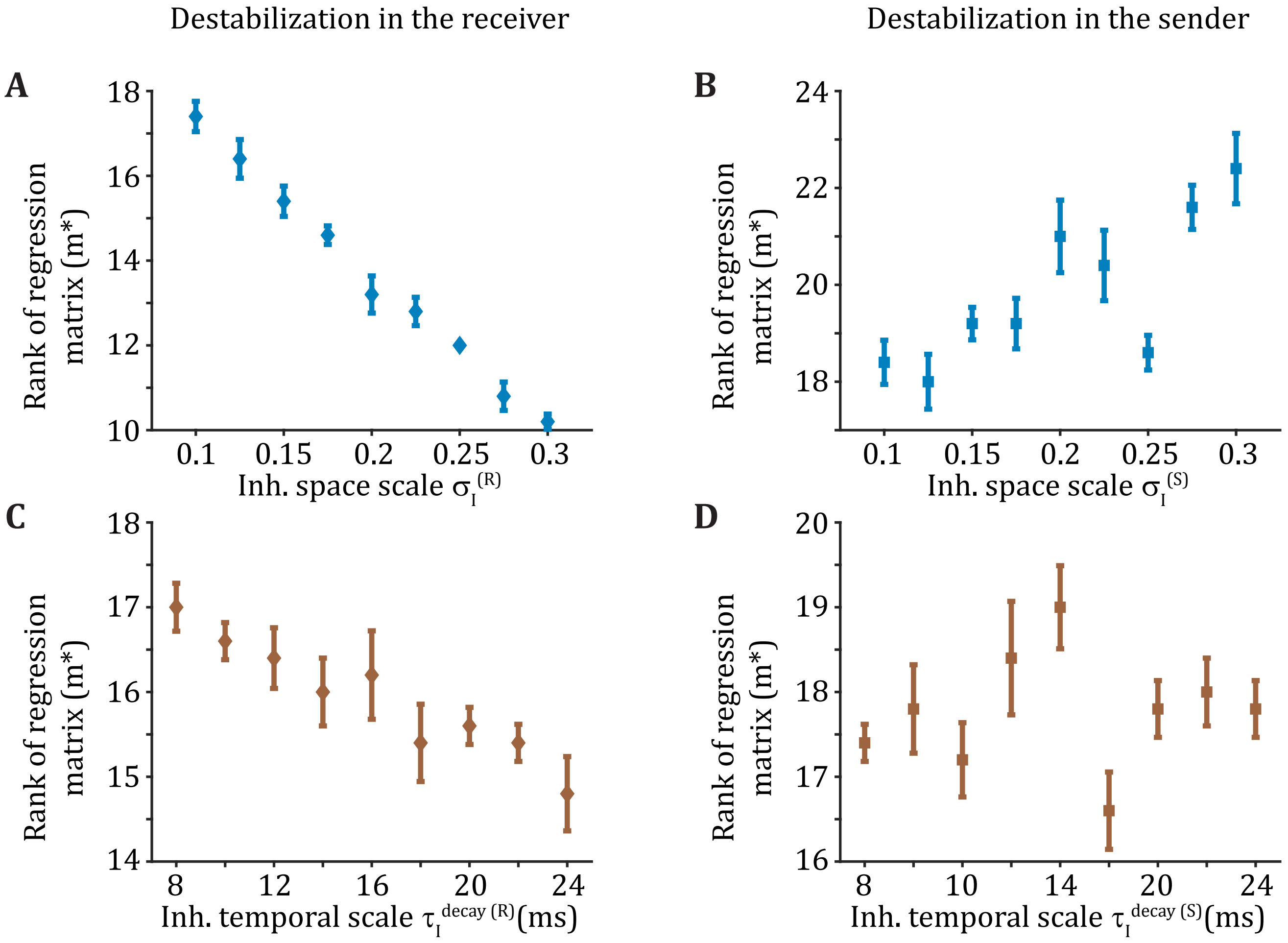

Figure S5: Optimal rank of the reduced-rank regression matrix.
(**A**) When the E/I balance is spatially destabilized by broadening the inhibitory space scale σ_I in the receiver, the optimal rank m^* of the reduced-rank regression, which corresponds to the dimension of the communication subspace, decreases. (**B**) When the E/I balance is spatially destabilized by increasing σ_I in the sender, the optimal rank m^* of the reduced-rank regression, which corresponds to the dimension of the communication subspace, increases. (**C,D**) Similar to (A,B) when the E/I balance is temporally destabilized by increasing the inhibitory timescale τ_I^{decay}.

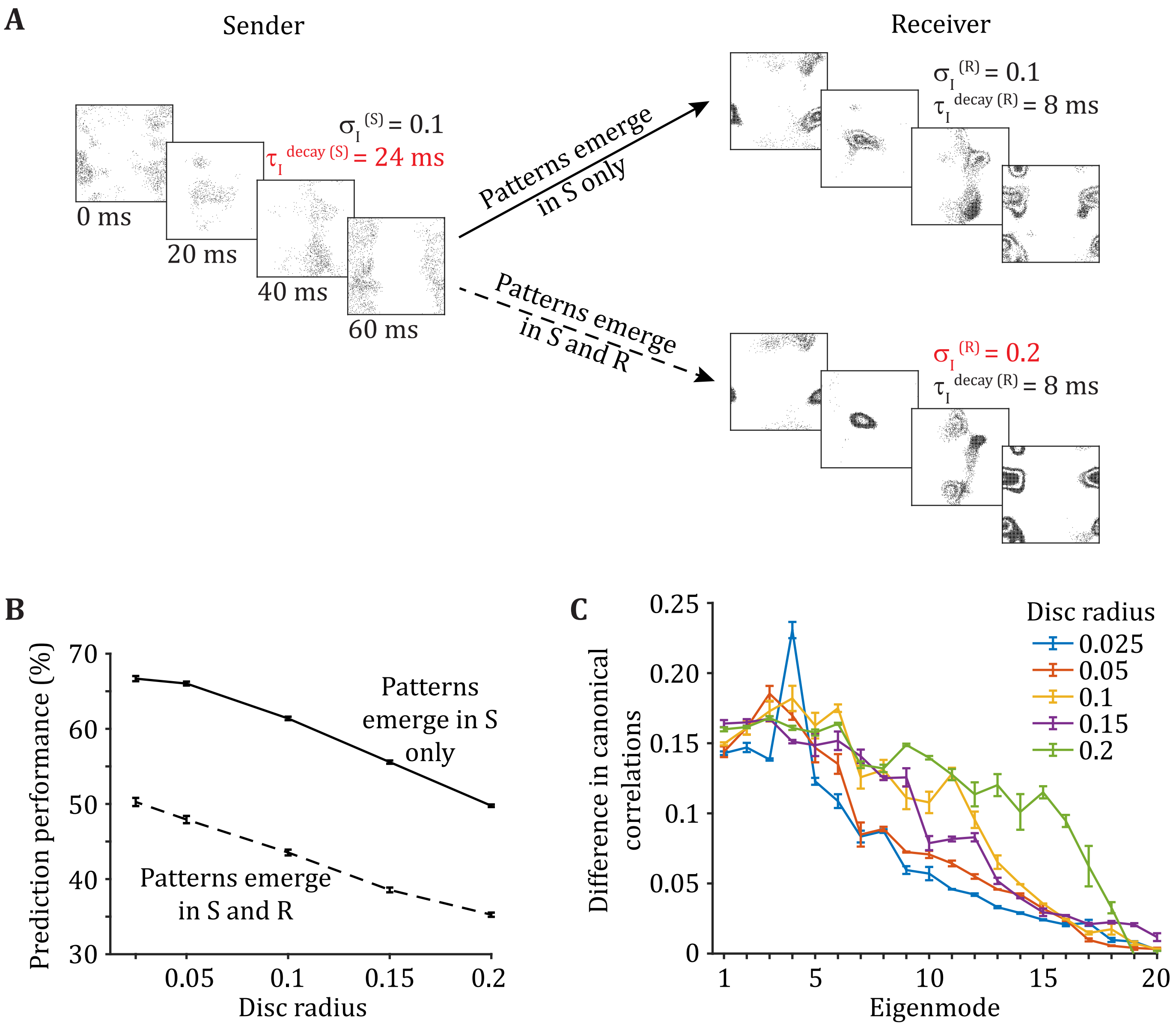

Figure S6: A misalignment of within-area shared variability is associated with poor communication between connected areas.
(**A**) The sender (S) is destabilized through a slower inhibitory timescale. The receiver (R) is either in the stable regime (top, patterns emerge in S only), or destabilized through broader recurrent inhibition (bottom, patterns emerge in S and R). By design, both networks have a good match in dimensionality of the sender and receiver networks (Fig. S7). (**B**) The prediction performance of the communication subspace between S and R is higher when only S is destabilized compared to when S and R are both destabilized. Therefore, dimensionality matching is not a sufficient condition for faithful communication. (**C**) Differences in canonical correlations between the case when only S is destabilized and the case when S and R are destabilized are positive for all disc radii (see Fig. S7 for the canonical correlations in each case individually). Hence, a misalignment of the within-area shared fluctuations in sender and receiver populations can cause poor communication.

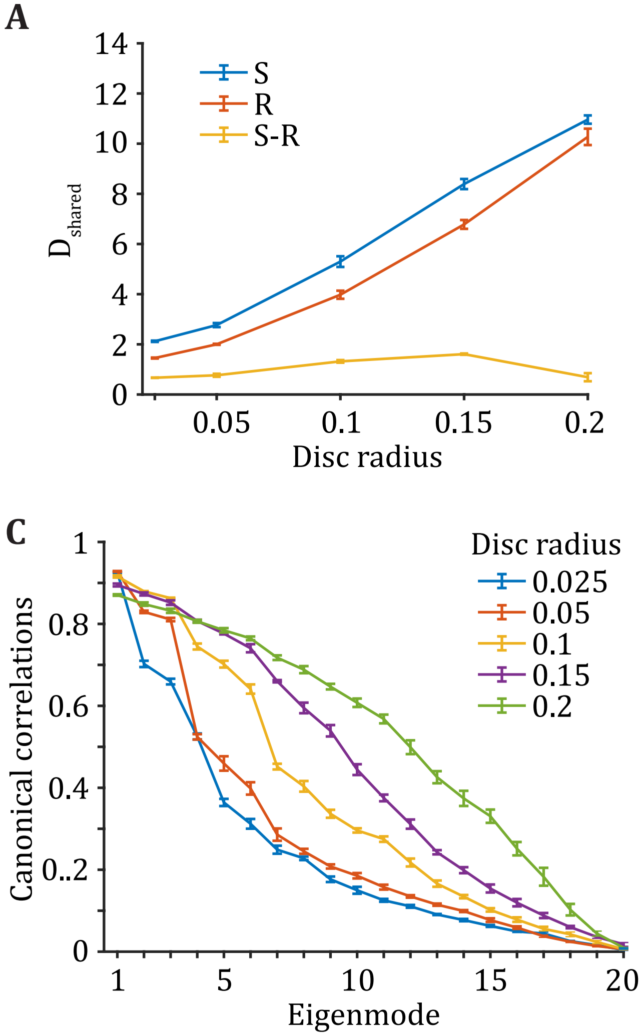

Figure S7: Emergence of novel spatio-temporal patterns in the receiver network induces a misalignment of the within-area shared variability in sending and receiving populations.

(**A**) Shared dimensionality D_{shared} in the sender network (S), the receiver network (R), and the difference between the two (S-R) in the case where spatio-temporal patterns emerge in S only (Fig. S6A, top). (**B**) Same as (A), except that different spatio-temporal patterns emerge in S and R (Fig. S6A, bottom). (**C,D**) Canonical correlations between the shared fluctuations in S and R when spatio-temporal patterns emerge in S only (C), and when different spatio-temporal patterns emerge in S and R (D).

REFERENCES AND NOTES

1. A. E. Urai, B. Doiron, A. M. Leifer, A. K. Churchland, Large-scale neural recordings call for new insights to link brain and behavior. *Nat. Neurosci.* **25**, 11–19 (2022).

2. M. L. Schölvinck, A. B. Saleem, A. Benucci, K. D. Harris, M. Carandini, Cortical state determines global variability and correlations in visual cortex. *J. Neurosci.* **35**, 170–178 (2015).

3. J. D. Murray, A. Bernacchia, N. A. Roy, C. Constantinidis, R. Romo, X.-J. Wang, Stable population coding for working memory coexists with heterogeneous neural dynamics in prefrontal cortex. *Proc. Natl. Acad. Sci. U.S.A.* **114**, 394–399 (2017).

4. R. C. Williamson, B. Doiron, M. A. Smith, B. M. Yu, Bridging large-scale neuronal recordings and large-scale network models using dimensionality reduction. *Curr. Opin. Neurobiol.* **55**, 40–47 (2019).

5. X. Jiang, H. Saggar, S. I. Ryu, K. V. Shenoy, J. C. Kao, Structure in neural activity during observed and executed movements is shared at the neural population level, not in single neurons. *Cell Rep.* **32**, 108006 (2020).

6. C. Langdon, M. Genkin, T. A. Engel, A unifying perspective on neural manifolds and circuits for cognition. *Nat. Rev. Neurosci.* **24**, 363–377 (2023).

7. N. A. Steinmetz, C. Koch, K. D. Harris, M. Carandini, Challenges and opportunities for large-scale electrophysiology with Neuropixels probes. *Curr. Opin. Neurobiol.* **50**, 92–100 (2018).

8. Y. Yu, J. N. Stirman, C. R. Dorsett, S. L. Smith, Mesoscale correlation structure with single cell resolution during visual coding. bioRxiv 469114 [Preprint] (2019). https://doi.org/10.1101/469114.

9. S. Musall, M. T. Kaufman, A. L. Juavinett, S. Gluf, A. K. Churchland, Single-trial neural dynamics are dominated by richly varied movements. *Nat. Neurosci.* **22**, 1677–1686 (2019).

10. M. R. Cohen, A. Kohn, Measuring and interpreting neuronal correlations. *Nat. Neurosci.* **14**, 811–819 (2011).

11. B. Doiron, A. Litwin-Kumar, R. Rosenbaum, G. K. Ocker, K. Josić, The mechanics of state-dependent neural correlations. *Nat. Neurosci.* **19**, 383–393 (2016).

12. M. Abeles, *Corticonics: Neural Circuits of the Cerebral Cortex* (Cambridge Univ. Press, 1991).

13. M. N. Shadlen, W. T. Newsome, The variable discharge of cortical neurons: Implications for connectivity, computation, and information coding. *J. Neurosci.* **18**, 3870–3896 (1998).

14. G. K. Ocker, Y. Hu, M. A. Buice, B. Doiron, K. Josić, R. Rosenbaum, E. Shea-Brown, From the statistics of connectivity to the statistics of spike times in neuronal networks. *Curr. Opin. Neurobiol.* **46**, 109–119 (2017).

15. A. Das, I. R. Fiete, Systematic errors in connectivity inferred from activity in strongly recurrent networks. *Nat. Neurosci.* **23**, 1286–1296 (2020).

16. J. P. Cunningham, B. M. Yu, Dimensionality reduction for large-scale neural recordings. *Nat. Neurosci.* **17**, 1500–1509 (2014).

17. J. D. Semedo, A. Zandvakili, C. K. Machens, B. M. Yu, A. Kohn, Cortical areas interact through a communication subspace. *Neuron* **102**, 249–259.e4 (2019).

18. R. Srinath, D. A. Ruff, M. R. Cohen, Attention improves information flow between neuronal populations without changing the communication subspace. *Curr. Biol.* **31**, 5299–5313.e4 (2021).

19. D. L. K. Yamins, J. J. DiCarlo, Using goal-driven deep learning models to understand sensory cortex. *Nat. Neurosci.* **19**, 356–365 (2016).

20. M. Diesmann, M.-O. Gewaltig, A. Aertsen, Stable propagation of synchronous spiking in cortical neural networks. *Nature* **402**, 529–533 (1999).

21. A. D. Reyes, Synchrony-dependent propagation of firing rate in iteratively constructed networks in vitro. *Nat. Neurosci.* **6**, 593–599 (2003).

22. A. Kumar, S. Rotter, A. Aertsen, Spiking activity propagation in neuronal networks: Reconciling different perspectives on neural coding. *Nat. Rev. Neurosci.* **11**, 615–627 (2010).

23. R. Rosenbaum, J. Trousdale, K. Josić, The effects of pooling on spike train correlations. *Front. Neurosci.* **5**, 58 (2011).

24. C. Van Vreeswijk, H. Sompolinsky, Chaotic balanced state in a model of cortical circuits. *Neural Comput.* **10**, 1321–1371 (1998).

25. D. J. Amit, N. Brunel, Model of global spontaneous activity and local structured activity during delay periods in the cerebral cortex. *Cereb. Cortex* **7**, 237–252 (1997).

26. A. Renart, J. de La Rocha, P. Bartho, L. Hollender, N. Parga, A. Reyes, K. D. Harris, The asynchronous state in cortical circuits. *Science* **327**, 587–590 (2010).

27. R. Rosenbaum, M. A. Smith, A. Kohn, J. E. Rubin, B. Doiron, The spatial structure of correlated neuronal variability. *Nat. Neurosci.* **20**, 107–114 (2017).

28. R. Darshan, C. van Vreeswijk, D. Hansel, Strength of correlations in strongly recurrent neuronal networks. *Phys. Rev. X* **8**, 031072 (2018).

29. I. D. Landau, H. Sompolinsky, Coherent chaos in a recurrent neural network with structured connectivity. *PLOS Comput. Biol.* **14**, e1006309 (2018).

30. F. Mastrogiuseppe, S. Ostojic, Linking connectivity, dynamics, and computations in low-rank recurrent neural networks. *Neuron* **99**, 609–623.e29 (2018).

31. A. Keane, P. Gong, Propagating waves can explain irregular neural dynamics. *J. Neurosci.* **35**, 1591–1605 (2015).

32. C. Huang, D. A. Ruff, R. Pyle, R. Rosenbaum, M. R. Cohen, B. Doiron, Circuit models of low-dimensional shared variability in cortical networks. *Neuron* **101**, 337–348.e4 (2019).

33. R. Chaudhuri, K. Knoblauch, M.-A. Gariel, H. Kennedy, X.-J. Wang, A large-scale circuit mechanism for hierarchical dynamical processing in the primate cortex. *Neuron* **88**, 419–431 (2015).

34. L. Muller, F. Chavane, J. Reynolds, T. J. Sejnowski, Cortical travelling waves: Mechanisms and computational principles. *Nat. Rev. Neurosci.* **19**, 255–268 (2018).

35. G. Hahn, A. Ponce-Alvarez, G. Deco, A. Aertsen, A. Kumar, Portraits of communication in neuronal networks. *Nat. Rev. Neurosci.* **20**, 117–127 (2019).

36. C. Holmgren, T. Harkany, B. Svennenfors, Y. Zilberter, Pyramidal cell communication within local networks in layer 2/3 of rat neocortex. *J. Physiol.* **551** (Pt 1), 139–153 (2003).

37. R. B. Levy, A. D. Reyes, Spatial profile of excitatory and inhibitory synaptic connectivity in mouse primary auditory cortex. *J. Neurosci.* **32**, 5609–5619 (2012).

38. L. F. Rossi, K. D. Harris, M. Carandini, Spatial connectivity matches direction selectivity in visual cortex. *Nature* **588**, 648–652 (2020).

39. R. Rosenbaum, B. Doiron, Balanced networks of spiking neurons with spatially dependent recurrent connections. *Phys. Rev. X* **4**, 021039 (2014).

40. C. Huang, Modulation of the dynamical state in cortical network models. *Curr. Opin. Neurobiol.* **70**, 43–50 (2021).

41. B. Everitt, *An Introduction to Latent Variable Models* (Chapman & Hall, 1984).

42. B. M. Yu, J. P. Cunningham, G. Santhanam, S. I. Ryu, K. V. Shenoy, M. Sahani, Gaussian-process factor analysis for low-dimensional single-trial analysis of neural population activity. *J. Neurophysiol.* **102**, 614–635 (2009).

43. L. Mazzucato, A. Fontanini, G. La Camera, Stimuli reduce the dimensionality of cortical activity. *Front. Syst. Neurosci.* **10**, 11 (2016).

44. A. Litwin-Kumar, K. D. Harris, R. Axel, H. Sompolinsky, L. F. Abbott, Optimal degrees of synaptic connectivity. *Neuron* **93**, 1153–1164 (2017).

45. V. Mante, D. Sussillo, K. V. Shenoy, W. T. Newsome, Context-dependent computation by recurrent dynamics in prefrontal cortex. *Nature* **503**, 78–84 (2013).

46. M. T. Kaufman, M. M. Churchland, S. I. Ryu, K. V. Shenoy, Cortical activity in the null space: Permitting preparation without movement. *Nat. Neurosci.* **17**, 440–448 (2014).

47. R. C. Williamson, B. R. Cowley, A. Litwin-Kumar, B. Doiron, A. Kohn, M. A. Smith, B. M. Yu, Scaling properties of dimensionality reduction for neural populations and network models. *PLOS Comput. Biol.* **12**, e1005141 (2016).

48. C. van Vreeswijk, H. Sompolinsky, Chaos in neuronal networks with balanced excitatory and inhibitory activity. *Science* **274**, 1724–1726 (1996).

49. M. London, A. Roth, L. Beeren, M. Häusser, P. E. Latham, Sensitivity to perturbations in vivo implies high noise and suggests rate coding in cortex. *Nature* **466**, 123–127 (2010).

50. M. Monteforte, F. Wolf, Dynamic flux tubes form reservoirs of stability in neuronal circuits. *Phys. Rev. X* **2**, 041007 (2012).

51. N. Mosheiff, B. Ermentrout, C. Huang, Chaotic dynamics in spatially distributed neuronal networks generate population-wide shared variability. *PLOS Comput. Biol.* **19**, e1010843 (2023).

52. K. Wimmer, A. Compte, A. Roxin, D. Peixoto, A. Renart, J. de la Rocha, Sensory integration dynamics in a hierarchical network explains choice probabilities in cortical area MT. *Nat. Commun.* **6**, 6177 (2015).

53. C. Gómez-Laberge, A. Smolyanskaya, J. J. Nassi, G. Kreiman, R. T. Born, Bottom-up and top-down input augment the variability of cortical neurons. *Neuron* **91**, 540–547 (2016).

54. C. Stringer, M. Michaelos, D. Tsyboulski, S. E. Lindo, M. Pachitariu, High-precision coding in visual cortex. *Cell* **184**, 2767–2778.e15 (2021).

55. C. Huang, A. Pouget, B. Doiron, Internally generated population activity in cortical networks hinders information transmission. *Sci. Adv.* **8**, eabg5244 (2022).

56. J. Barbosa, R. Proville, C. C. Rodgers, M. R. DeWeese, S. Ostojic, Y. Boubenec, Early selection of task-relevant features through population gating. *Nat. Commun.* **14**, 6837 (2023).

57. X.-J. Wang, Probabilistic decision making by slow reverberation in cortical circuits. *Neuron* **36**, 955–968 (2002).

58. A. H. Lara, J. P. Cunningham, M. M. Churchland, Different population dynamics in the supplementary motor area and motor cortex during reaching. *Nat. Commun.* **9**, 2754 (2018).

59. G. Chen, B. Kang, J. Lindsey, S. Druckmann, N. Li, Modularity and robustness of frontal cortical networks. *Cell* **184**, 3717–3730.e24 (2021).

60. R. Pyle, R. Rosenbaum, Spatiotemporal dynamics and reliable computations in recurrent spiking neural networks. *Phys. Rev. Lett.* **118**, 018103 (2017).

61. J. D. Semedo, A. I. Jasper, A. Zandvakili, A. Krishna, A. Aschner, C. K. Machens, A. Kohn, B. M. Yu, Feedforward and feedback interactions between visual cortical areas use different population activity patterns. *Nat. Commun.* **13**, 1099 (2022).

62. J. A. Gallego, M. G. Perich, R. H. Chowdhury, S. A. Solla, L. E. Miller, Long-term stability of cortical population dynamics underlying consistent behavior. *Nat. Neurosci.* **23**, 260–270 (2020).
